# Scientific publications in ophthalmic journals from China and other top-ranking countries: a 12-year review of the literature

**DOI:** 10.1186/1471-2415-13-25

**Published:** 2013-06-26

**Authors:** Wenbin Huang, Wei Wang, Jiao Zhan, Minwen Zhou, Shida Chen, Xiulan Zhang

**Affiliations:** 1 Zhongshan Ophthalmic Center, State Key Laboratory of Ophthalmology, Sun Yat-Sen University, Guangzhou, People’s Republic of China

**Keywords:** Bibliometrics, Ophthalmology, Publication, China, Top-ranking countries

## Abstract

**Background:**

Eye diseases with increasing mortality are common health problems that affect people of all ages and demographic backgrounds. In this study, we study the publication characteristics in international ophthalmic journals of the US, the UK, Germany, Australia, Japan, and China.

**Methods:**

Articles published in 53 ophthalmic journals from 2000 to 2011 were retrieved from the PubMed database. We recorded the number of articles published each year, analyzed the publication type, and evaluated the accumulated and average impact factors (IFs), and the distribution of articles in ophthalmic journals in relation to IFs. The characteristics of publication outputs from China and other top-ranking countries were compared.

**Results:**

The total number of articles increased significantly during the past 12 years, with an increase of 51.0%. The growth in the annual number of articles from the US, the UK, Australia, and China showed a significantly positive trend. Publications from the US exceeded those from any other country and had the highest IFs, largest number of total citations of articles, and the most articles published in leading ophthalmic journals. During the past 12 years, China contributed 3.5% of the total publications, and the number of Chinese articles showed a more than 6-fold increase (from 99 to 605, R2 =0.947, P<0.001). The numbers of IFs and citations of articles originating in China were mostly lower than for other top-ranking counties.

**Conclusions:**

Research on ophthalmic journals has maintained an upward growing trend from 2000 to 2011. Chinese ophthalmology research has developed rapidly, but the gap still exists between China and other top-ranking countries for the advanced level of research.

## Background

Scientific publications, providing a link between the production of knowledge and its use, is one recognized way to measure academic achievement and serve an important role in the scientific process [1]. Thus, many bibliometric researches had been devoted to the field of medicine, such as oncology [2], anesthesiology [3], gastroenterology [4], and ophthalmology [5] et al.

Eye diseases are common health problems that affect people of all ages and demographic backgrounds. The current estimate for blindness worldwide currently stands at 45 million persons, and this number increases by 1–2 million each year [6]. Researchers and scientific communities of different countries have gradually come to pay more attention to this condition, and are now attempting to understand the disease pathogenesis and to develop new diagnostic methods and remedies. However, to date, little is known about the relative contributions of China and other top-ranking countries to the field of ophthalmology. The lack of objective information about current research output creates difficulty when endeavoring to plan for necessary improvements in infrastructure related to the understanding, treatment, and prevention of eye diseases. Thus, accurate assessment of global and regional productivity in ongoing research in ophthalmology is important. The present study was designed to study the publication characteristics in international ophthalmic journals of China and other top-ranking countries over the past 12 years (2000.1.1-2011.12.31).

## Methods

In this study, a total of 53 ophthalmology journals were selected. The selection criteria as previous studies described [2,7,8] was that the journal (i) was listed in the “ophthalmology” category of Science Citation Index Expanded (SCIE) subject categories by the Institute for Scientific Information (ISI); (ii) was indexed in the PubMed database; and (iii) had impact factors (IFs) according to Thomson Reuters in ISI’s 2011 Journal Citation Reports (JCR) [9].

The PubMed database was searched within a specific date range (2000.1.1 and 2011.12.31). The International Standard Serial Numbering (ISSN) (Print) was used to perform searches in PubMed. For ISSN (Print) of the top 10% ophthalmology journals with IF scores, the following search terms were used: “1350-9462 OR 0161–6420 OR 0002–9394 OR 1542–0124 OR 0003-9950”. The five ISSN (Print), in turn, represented the following journals: “*Prog Retin Eye Res*”, “*Ophthalmology*”, “*Am J Ophthalmol*”, “*Ocul Surf*”, and “*Arch Ophthalmol*”. The information within all selected articles was drawn out independently by two investigators (WB Huang and W Wang), who surveyed the titles, authors, abstracts, publication types, and other details. Discrepancies were resolved by review of the full text. The research output from different countries was determined using the first author’s institutional affiliations. IFs from 2000 to 2011 were determined using (JCR). In addition, the number of citations of every article was calculated using the Web of Science of ISI Database.

Three methods were used to compare publication characteristics. First, the total annual number of articles related to ophthalmology was calculated. Second, the publication types of the articles were analyzed. Original clinical trials (including cross-sectional and prospective cohort studies), randomized controlled trials (RCT), and case reports were compiled using the publication type categories of the PubMed database. Third, the IF, a measure reflecting the average number of citations to recent articles published in the journal, is usually used for measuring and comparing the influence of entire journals. In this study, the accumulated and average IFs, citations of every article, and the distribution of articles in ophthalmic journals in relation to IFs were calculated to permit comparison of the quality of the publications as previous studies described [2,7]. These were mainly from the top-ranking countries ranked by the total number of articles produced during the past 12 years.

Statistical analyses were performed using SPSS 20.0 software (IBM, New York, US), and statistical results are given in Tables and Figures. The Kruskal-Wallis test and the Mann–Whitney test were used to detect differences between countries. The trends with respect to number of articles were analyzed via curvilinear regression. Significance was tested using the two-tailed test, and the value of p < 0.05 was considered significant.

## Results

### Total number of articles

**Figure 1:**
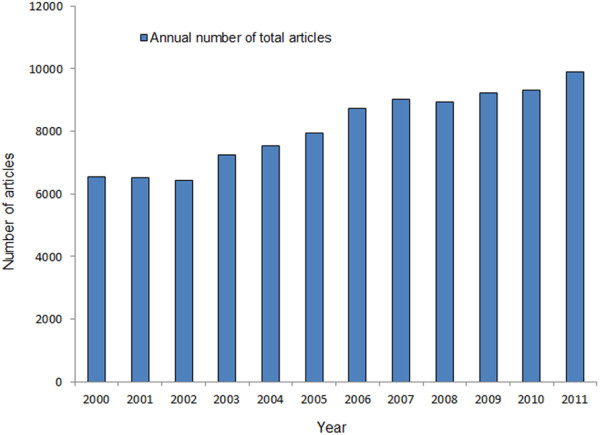
Number of articles published in journals worldwide during the past 12 years.

**Figure 2:**
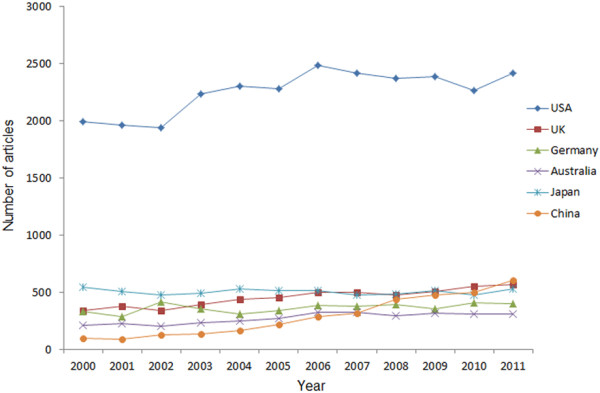
Trends in annual numbers of articles written by researchers from the six countries from 2000 to 2011.

Between 2000 and 2011, a total of 97326 articles were published in the 53 selected worldwide journals indexed in PubMed. The number of published articles in the fields of ophthalmology around the world increased significantly from 2000 to 2011 (R^2^ = 0.951, P < 0.001), with an increase of 51.0% (from 6556 articles in 2000 to 9898 in 2011) (Figure 1). The top five countries ranked by number of articles were the US (27.8%, 27034 /97326), Japan (6.2%, 6045/97326), the UK (5.6%, 5432/97326), Germany (4.5%, 4355/97326), and China (3.5%, 3446/97326) (this included mainland China (1761), Hong Kong (762), and Taiwan (923)). Australia (3.4%, 3278/97326) ranked the sixth. The six countries together accounted for 51.0% of total number of all articles. The growth in the annual number of published articles from the US, the UK, Australia, and China increased significantly (the US: 1989 to 2415, R^2^ =0.612, P=0.003; the UK: 336 to 566, R^2^ =0.925, P<0.001; Australia: 214 to 307, R^2^ =0.769, P<0.001; China: 99 to 605, R^2^ =0.947, P<0.001) (Figure 2). In contrast, the annual number publications from Germany and Japan did not change significantly.

### Clinical trials, randomized controlled trials, and case reports

**Figure 3:**
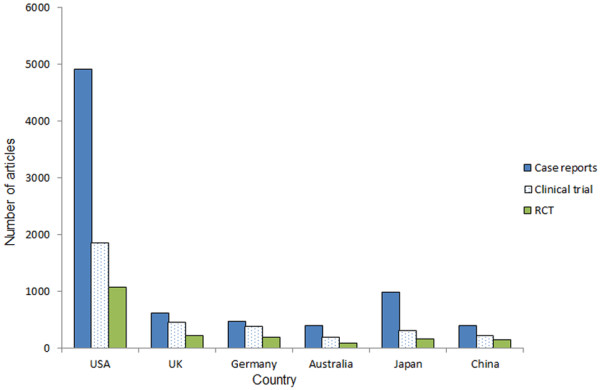
Number of case reports, clinical trials, and RCTs written by authors from the six countries from 2000 to 2011.

There were 18609 case reports, 6838 clinical trials, and 3781 RCTs published from 2000 to 2011. Of all the 97326 articles published in that period, case studies accounted for 19.1%, and researchers from the US and Japan made the main contributions. Authors from the US published the most case reports (4900, 26.3% of total), clinical trials (1858, 27.2% of total), and RCTs (1066, 28.2% of total). The ranking of the other five countries with respect to the number of clinical trials or RCTs was: the UK (466 clinical trials, 226 RCTs), Germany (392 clinical trials, 197 RCTs), Japan (320 clinical trials, 159 RCTs), China (236 clinical trials, 139 RCTs), and Australia (209 clinical trials, 86 RCTs) (Figure 3).

### Impact factors

**Figure 4:**
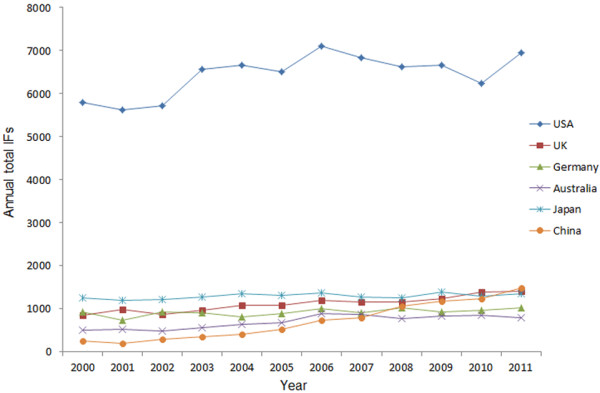
Annual total IFs of articles published by authors from the top six countries between 2000 and 2011.

In accordance with the JCR 2011, we calculated the accumulated and average IFs for the six countries. The annual total IFs (Figure 4) were similar to the results shown in Figure 1. The annual total of IFs from the US was the highest, and there were significant differences among these six countries (P<0.001). China showed significantly positive trends in the period 2000–2011 (R^2^ =0.950, P<0.001). In addition, the average IFs results were shown in Table 1. The average IFs from the US were significantly higher than others (p<0.001), while the results from other five countries differed insignificantly (p=0.104).

**Table 1:** Average impact factors of articles written by authors from six countries from 2000 to 2011

**Year**	**USA**	**UK**	**Germany**	**Australia**	**Japan**	**China**
2000	2.911	2.556	2.780	2.369	2.312	2.561
2001	2.869	2.640	2.507	2.352	2.350	2.186
2002	2.950	2.522	2.230	2.340	2.545	2.383
2003	2.936	2.438	2.571	2.425	2.587	2.541
2004	2.891	2.501	2.594	2.548	2.547	2.450
2005	2.857	2.401	2.620	2.473	2.531	2.380
2006	2.864	2.393	2.563	2.705	2.664	2.608
2007	2.829	2.323	2.391	2.704	2.694	2.486
2008	2.798	2.428	2.592	2.637	2.605	2.446
2009	2.794	2.449	2.630	2.627	2.709	2.490
2010	2.756	2.523	2.347	2.695	2.686	2.490
2011	2.874	2.473	2.554	2.608	2.564	2.444
Total	2.856	2.460	2.525	2.567	2.569	2.471

The distribution of articles from different countries, in relation to IFs, is shown in Table 2. Between 2000 and 2011, a total of 8592 articles were published in the top 10% journals with IFs. Of these, 54.2% (6096/8592) originated in the USA, 9.2% (1041/8592) in Japan, and 3.4% (378/8592) in China. In total, 24007 articles were published in the top 25% journals: 39.0% of the articles came from the US, 5.7% from the UK, 4.3% from Germany, 3.3% from Australia, 7.3% from Japan, and only 3.1% from China. In the top 50% of journals with IFs upon 1.561, the six countries accounted for 58.1% of all articles; while in the bottom 50% of journals, the percentage was 34.1%.

**Table 2:** Distribution of articles in ophthalmic journals in relation to impact factors from the top five countries and China during 2000 and 2011

**Impact factors range**	**Articles number and percentage (n (%))**						**Total (n)**	**Impact factors**	**Journal number**
	**USA**	**UK**	**Germany**	**Australia**	**Japan**	**China**			
Top 10%	6096 (54.2)	275 (2.4)	392 (3.5)	411 (3.7)	1041 (9.2)	378 (3.4)	8592	9.455-3.711	5
Top 25%	14923 (39.0)	2189 (5.7)	1656 (4.3)	1262 (3.3)	2774 (7.3)	1203 (3.1)	24007	9.455-2.629	13
Top 50%	21803 (31.4)	4866 (7.0)	3698 (5.3)	2679 (3.9)	4497 (6.5)	2774 (4.0)	40318	9.455-1.561	27
Bottom 50%	5231 (19.2)	566 (2.1)	657 (2.4)	599 (2.2)	1548 (5.7)	672 (2.5)	9272	1.509-0.129	26
Bottom 25%	2247 (15.8)	145 (1.0)	280 (2.0)	86 (0.6)	986 (6.9)	111 (0.8)	3853	0.926-0.129	13
Bottom 10%	34 (0.6)	51 (1.0)	107 (2.0)	1 (0.0)	4 (0.1)	0 (0.0)	196	0.510-0.129	5

Data do not sum up to 100% because percentage of other countries is not included. “Total” means the world’s output in the relative journals in the period 2000–2011.

### Citations

**Figure 5:**
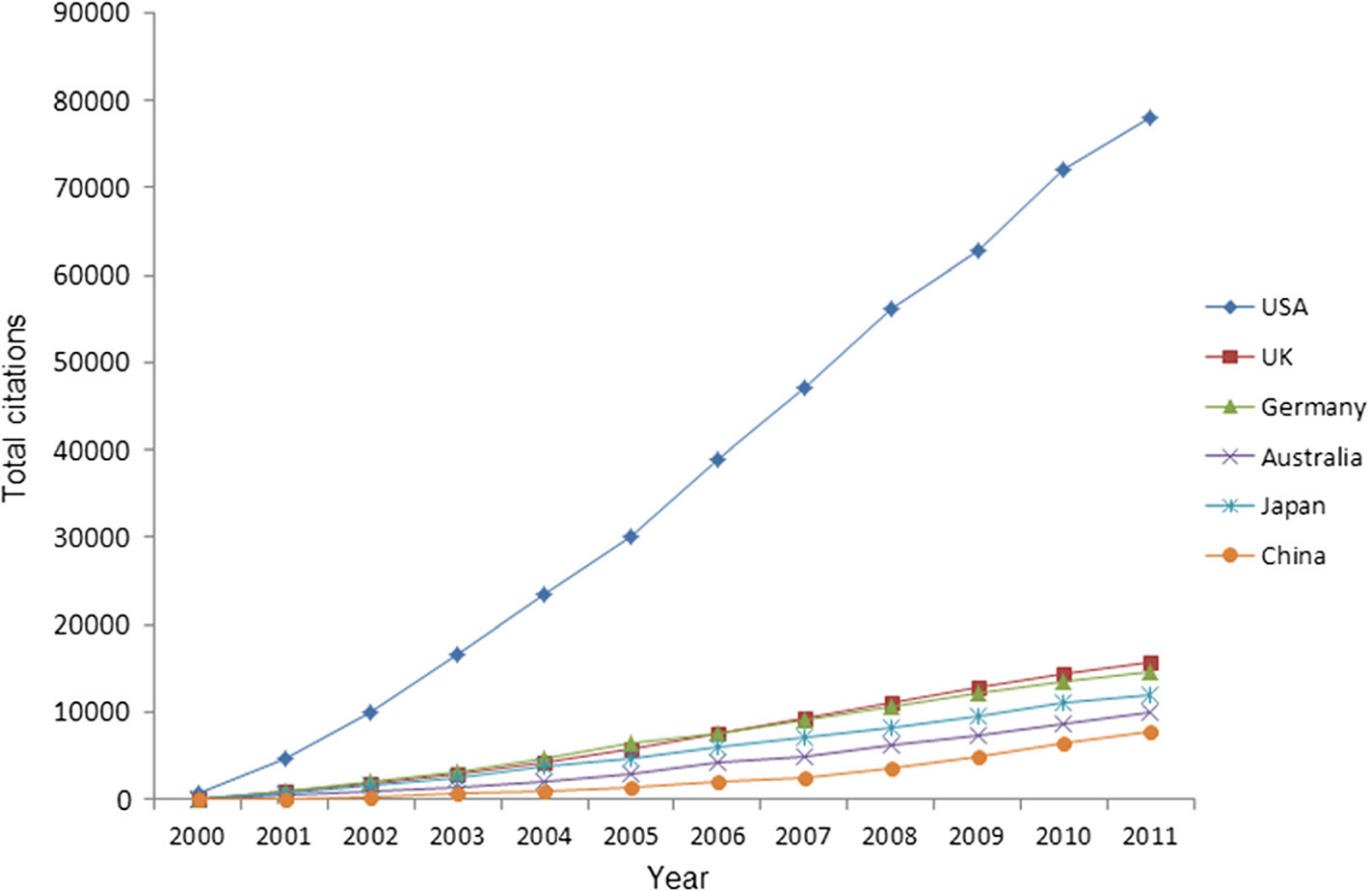
Annual citations of articles written by authors from China and other top-ranking countries from 2000 to 2011.

**Figure 6:**
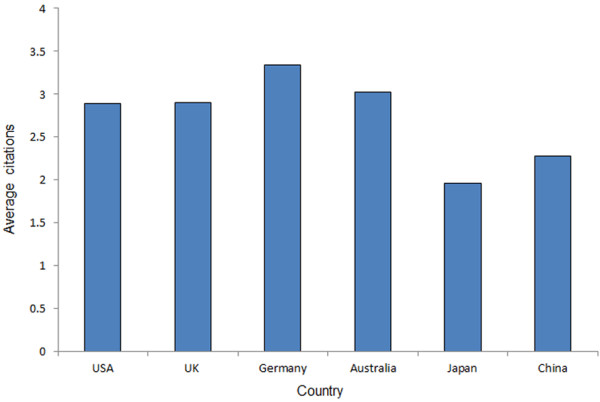
Average number of citation in articles published by authors from the six countries during the past 12 years.

Using the ISI database, we analyzed the total number of citations and the average citation rate from China and other top-ranking countries. The results of the total number of citations are shown in Figure 5. From 2000 to 2011, The US had 27034 articles and 78093 citations ranking the first, followed by the UK (5432 articles, 15729 citations), Germany (4355 articles, 14548 citations), Australia (3278 articles, 9893 citations) and Japan (6045 articles, 11861 citations). China had 3446 articles and 7859 citations, putting it at the lowest ranking among these six countries. These differences among these six countries were found to be significant (P<0.001). Papers from Germany had the greatest average number of citations (3.34), followed by Australia (3.02), the UK (2.90), the US (2.89), and China (2.28) (Figure 6). Japanese papers had the fewest citations on average when compared to papers from the other five countries.

### Favorite ophthalmic journals

The most popular journals are listed in Table 3. *Invest Ophth Vis Sci* (8866) was the No. 1 journal with respect to number of total published articles from 2000 to 2011, followed by *Brit J Ophthalmol* (5781), *J Cataract Refr Surg* (5661), *Ophthalmology* (5564), and *Am J Ophthalmol* (5414). The favorite journal for the US, the UK, Germany, Australia, Japan, and China was *Invest Ophth Vis Sci, Brit J Ophthalmol, Graef Arch Clin Exp, Clin Exp Ophthalmol, Jpn J Ophthalmol,* and *Mol Vis*, respectively.

**Table 3:** The five most popular journals for researchers from China and other top-ranking countries between 2000 and 2011

**Rank**	**Total**	**USA**	**UK**	**Germany**	**Australia**	**Japan**	**China**
1	Invest Ophth Vis Sci (8866)	Invest Ophth Vis Sci (4151)	Brit J Ophthalmol (1204)	Graef Arch Clin Exp (922)	Clin Exp Ophthalmol (550)	Jpn J Ophthalmol (959)	Mol Vis (433)
2	Brit J Ophthalmol (5781)	Am J Ophthalmol (2242)	Eye (1200)	Invest Ophth Vis Sci (507)	Invest Ophth Vis Sci (399)	Invest Ophth Vis Sci (792)	Invest Ophth Vis Sci (412)
3	J Cataract Refr Surg (5661)	Ophthalmology (2083)	Vision Res (565)	Vision Res (343)	Clin Exp Optom (341)	Am J Ophthalmol (674)	Eye (252)
4	Ophthalmology (5564)	Arch Ophthalmol (1870)	Invest Ophth Vis Sci (375)	Brit J Ophthalmol (310)	Brit J Ophthalmol (245)	Graef Arch Clin Exp (395)	Graef Arch Clin Exp (230)
5	Am J Ophthalmol (5414)	Exp Eye Res (1435)	Ophthal Physl Opt (341)	J Cataract Refr Surg (283)	Vision Res (240)	Brit J Ophthalmol (353)	Ophthalmology (188)

## Discussion

To the best of our knowledge, this is the first report to analyze the distribution of ophthalmic papers from 2000 to 2011, and it is also the first study to evaluate the publication characteristics of China and other top-ranking countries.

In general, the number and percentage of research articles published in scientific journals is a reflection of research activity within a country [10]. The total number of articles published in ophthalmic journals around the world maintained an upward growing trend from 2000 to 2011, with an increase of 51.0%, Which, to some degree, may due to increasing number of ophthalmic journals (from 46 to 53 between 2000 and 2011) and the enlarged capacity of journals. The trends in the annual number of articles from the US, the UK, Australia, and especially China showed clear increases (P<0.05), suggesting that these countries contributed more to the developing of ophthalmology between 2000 and 2011. On the other hand, the increase in rate for Germany and Japan was not considerable, probably because the research on ophthalmology has reached a bottleneck or has encountered some kind of limiting factor. The differences of publications among countries may be socioeconomic related, for example, number of investigators, research fund, number of ophthalmologists et al. In addition, the present study shows that the percentage of the world output in ophthalmology literature is the highest for the US (accounting for 27.8% of the total). There is no doubt that the US has a great deal of international influence on scientific research and that it leads the field of scientific research [2,7,11], which also applies to ophthalmology. Huge amounts of research funding and an abundance of trained researchers might explain the fact that the US has achieved a state of continuous dominance.

Only 3781 RCTs were found among all 97362 articles and the US accounted for a considerable proportion of these. Researchers from the other five countries published relatively few RCTs. Overall, RCTs are considered to be the most reliable form of scientific evidence in the hierarchy of evidence that influences healthcare policy and practice. Researchers should therefore put more emphasis on RCTs and consider these findings. During the past 12 years 199 meta-analyses were published, which mainly evaluated the effects of therapy, risk factors, diagnostic criteria, or prevalence. On the other hand, the importance of basic research should not be discounted. The medical research chain needs scientists, clinician-scientists, and expert clinicians to succeed in both the basic and clinical research environments [12].

The IF is frequently used as a proxy for the relative importance of a journal within its field, with journals with higher IFs deemed to be more important than those with lower ones. Researchers from the US have published more papers in high IFs journals, with the highest annual total and average IFs, which revealed that they contributed the most to ophthalmic research with respect to both quantity and quality. No significant differences were found in the average IFs among the UK, Germany, Australia, Japan, and China during the past 12 years. On the other hand, the distribution of articles from different countries in relation to journal IFs (Table 2) showed that these five developed countries (the US, the UK, Germany, Australia, and Japan) and China accounted for over 50% in the both top 10%, 25%, and 50% journals. This suggested that these six countries were at the forefront of global scientific research in the ophthalmology field.

Citations are another indicator of publication quality, as they indicate the degree to which the paper has been accepted by other authors in the same field. Articles from the US had fairly high total numbers of citations, followed by the UK, Germany, Japan, Australia, and China. However, the average number of citations from the US ranked the fourth, behind Germany, Australia and the UK. This may reflect the possibility that document type can affect citation, and that the US published the most case reports that are rarely cited [13]. Articles from China ranked low in terms of cumulative or average number of citations during the past 12 years. The quality of articles from China is probably not high and needs to be improved.

In the present study, *Invest Ophth Vis Sci* was found to be the most popular journal. *Invest Ophth Vis Sci*, published online several times a month, is an official journal of the Association for Research in Vision and Ophthalmology (ARVO). It mainly publishes high quality basic research articles, which leads to its popular international impact. However, the most popular journal for authors differed among the six top-ranking countries. A regional journal preference trend was evident; for example, researchers from the UK publish most in *Brit J Ophthalmol*, which may be due to the UK publications are in journals with offices in the UK. Similar trend is shown by the authors for US (*Invest Ophth Vis Sci*), Germany (*Graef Arch Clin Exp*), Australia (*Clin Exp Ophthalmol*), Japan (*Jpn J Ophthalmol*) and China (*Mol Vis*).

In the past 12 years, the absolute number of Chinese articles showed a more than 6-fold increase (from 99 to 605 between 2000 and 2011) in publications in international ophthalmic journals, which was a significant positive trend (R^2^ =0.947, P<0.001). In 2011, articles originating in China surpassed the UK, which ranked second (Figure 2). In the last decade, the tremendous economic growth of China has propelled the world’s most populous country to the forefront of the global economy and politics. Research and development funds provided by the Chinese government have increased rapidly. With this type of major socioeconomic impetus, a marked development in science and medicine has also inevitably taken place [7]. Moreover, in recent years, increasingly more scientists with notable achievements in ophthalmic research have come to China from the US and Europe and are now actively involved in the promotion of ophthalmic research in China. However, the analysis of the publication types revealed that few papers on RCTs have been written by Chinese researchers. In addition, as in many other developing countries, China has experienced dramatic demographic and epidemiological transitions. Its elderly population is increasing rapidly; thus, the morbidity of age-related eye diseases is growing accordingly. Therefore, Chinese researchers should launch more multicenter prospective studies and RCTs aimed at risk factors, diagnosis, treatments, and prevention. In addition, the numbers of IFs and citations of articles originating in China were lower than for other top-ranking counties. The quality of articles and the international influence from China also needs to be improved.

There are some limitations to this study. Firstly, the number of publications identified from these ophthalmic journals is only an estimation of the total publications. In the field of ophthalmology, many important articles are published in other non-ophthalmic journals. Secondly, the six countries selected may not represent the status of all articles and may also not be representative of the international level of ophthalmic research. However, these countries have produced in excess of 50% of the total number of articles published during the past 12 years, which suggests that research in these countries is advanced. Thirdly, we only included English-language journals, which might have resulted in an overrepresentation of English-speaking countries. This is because English-speaking countries have more English journals than others, and English-writing is easier for them. Writing English-language articles is not always common practice in China. A substantial number of articles are published in local journals. Fourthly, for some studies conducted in joint collaboration with other regions or countries, only the first authors’ affiliations are included as the origin of research in the PubMed database, which neglects the contributions of other researchers from different geographic areas. Finally, although the IF can be used as a rough indictor, it still has many limitations [14,15]. However, the IF is still widely regarded as the best instrument for the evaluation of the quality of scientific journals [15].

## Conclusions

In conclusion, the present study provided some useful information about scientific studies of ophthalmology. The US has led the productivity of ophthalmology research in the past 12 years. Chinese ophthalmology research has developed rapidly, but the gap between China and other top-ranking countries at the advanced levels of ophthalmology research is still enormous. Chinese ophthalmologists need to improve their research activity and to gear up for high-quality studies.

## Competing interests

The authors declare that they have no competing interests.

## Authors’ contributions

All authors conceived of and designed the experimental protocol. WBH and WW collected the data. All authors were involved in the analysis. WBH wrote the first draft of the manuscript. WBH, WW, JZ and XLZ reviewed and revised the manuscript and produced the final version. All authors read and approved the final manuscript.

## Pre-publication history

The pre-publication history for this paper can be accessed here:

http://www.biomedcentral.com/1471-2415/13/25/prepub
